# Expression patterns of *kiss2* and *gpr54‐2* in *Monopterus albus* suggest these genes may play a role in sex reversal in fish

**DOI:** 10.1002/2211-5463.12727

**Published:** 2019-09-10

**Authors:** Ti‐Lin Yi, Meng‐Ting Pei, Dai‐Qin Yang

**Affiliations:** ^1^ Yangtze University Engineering Research Center for Ecology and Agriculture Use of Wetland, Ministry of Education Jingzhou China; ^2^ School of Animal Science Yangtze University Jingzhou China; ^3^ Hubei Provincial Engineering and Technology Research Center for Monopterus albus Jingzhou China; ^4^ Hubei Zhongqing Aquaculture Industry Technology Research Institute and limited company Jingzhou 434026 China

**Keywords:** Asian swamp eel, gene expression, HPG axis, Kisspeptin/GPR54 system

## Abstract

Due to its exceptionally small genome size and protogynous hermaphroditism, *Monopterus albus* has been proposed as a model for vertebrate sexual development. The Kiss/GPR54 system is a central regulator of sexual development in most vertebrates, but its role in sex reversal remains hypothetical. In contrast to mammals, fishes often possess more than one copy of the *kiss* and *gpr54* genes. Our objectives were to identify all *kiss*/*gpr54* genes in the genome of *M. albus* and to assess their involvement in sex reversal via their expression patterns (qPCR) in females, males, and intersex specimens. We identified only two genes: *kiss2* and *gpr54‐2*. *kiss2* expression was extremely high in the gonads of males, intermediate in females, and low in intersex; and reduced in all tissues of intersex. *gpr54* expression was also extremely high in the gonads of males, high in intersex, but low in females. *gpr54* expression in brain was high in all three sexes. In conclusion, (a) *kiss1* has been functionally replaced in *M. albus*; (b) the functions of *gpr54‐2* in brain are not sex‐specific; (c) *kiss2* appears to undergo a ‘reset’ in the expression during the sex change; and (d) sex‐specific expression patterns in the gonads indicate that these two genes may play a role in sex reversal in fish.

**Abbreviations**
HPG: hypothalamus–pituitary–gonadal

Whereas the sexual fate of a majority of vertebrates is irreversibly determined during embryonic development, many fish species exhibit remarkable sexual plasticity 1. In some species, this plasticity manifests itself in an intriguing phenomenon of sex reversal during their adult life 1, 2. Among these is a freshwater teleost species Asian swamp eel *Monopterus albus* (Zuiew, 1793; Synbranchiformes: Synbranchidae) native to South‐East Asia. It is protogynous: It starts its sexual life history as a female, undergoes an intersex stage, and then develops into the final male stage 3, 4. Although sex reversal of this fish has received some scientific attention in recent years, for example, 3, 5, 6, the control and molecular mechanisms of this process remain poorly understood. As this species also has an exceptionally small genome, it has been proposed as a model system for sexual development in vertebrates 3, 4. It is also an economically important cultured fish in many Asian countries 7, but the sex reversal causes low fecundity and presents a major obstacle for large‐scale breeding 8. Therefore, our research unit is currently undertaking a research project that aims to improve our understanding of the molecular control of sex reversal in *M. albus*.

KisspeptinGPR54/system is a component of the hypothalamus–pituitary–gonadal (HPG) axis with a central role in the development of sexual maturity (and sex reversal) in vertebrates. Precisely, Kisspeptin interacts with GPR54 to regulate the secretion of gonadotropin‐releasing hormone from hypothalamus 9. The ancestral state for vertebrates is believed to be the existence of two *kiss* (*kiss1* and *kiss2*) genes (encoding the Kisspeptin protein) and two Kisspeptin receptors (*gpr54*, also known as *kissr* gene), but the *kiss2* system was lost in all mammals 10. As opposed to this, in fish, the number of paralogues of *kiss* and *gpr54* varies from only one to several (four) 10, 11, 12, 13. As there is evidence that *kiss1 and kiss2* paralogues have different functions 12, 14, 15, this variability in the number of *kiss* genes is rather intriguing.

Although fishes possess a number of characteristics that make them a suitable model for studying the Kisspeptin/GPR54 system 16, 17, the complexity of the system, the existence of compensatory mechanisms, and species specificity of the system hamper the progress of these studies, so the exact roles of kisspeptin signaling genes in sexual development in fish remain debated 18, 19. Whereas the Kisspeptin/GPR54 expression has been studied extensively in fish in the context of brain–pituitary axis, early development, onset of puberty, spawning cycles, and annual gonadal stages 18, 20, there appears to be only one report of the expression of these genes in the context of sex reversal 12. As a result, although the association between Kisspeptin signaling and sex reversal has been proposed 1, 12, the role of this system in sex reversal remains unknown.

As Kisspeptin system has not been studied in *M. albus* at all, the objectives of this study were as follows: (a) to identify all extant *kiss* and *gpr54* genes in the genome of *M. albus*; (b) to indirectly corroborate their roles in the sex reversal in this fish by studying their expression patterns in different tissues of male, female, and intersex (undergoing the sex reversal) specimens.

## Methods

### Identification and characterization of *kiss* and *gpr54* genes

We relied on the published (GenBank Acc. No. AONE00000000) complete genome of *M. albus* 4, and transcriptome data for different developmental stages (accession number SRX1007627) 5, to identify all extant *kiss* and *gpr54* genes (including paralogues) using BLASTn suite. To identify any putative splice variant isoforms as well 10, we searched proteomes of three gonad types during sex reversal 21. To indirectly corroborate the identity of the two genes, we conducted phylogenetic analyses using datasets comprising 25–30 vertebrate homologues (we selected four different major taxa: fish, amphibians, mammals, and Monotremata; GenBank accession numbers are shown in the figures). For this, we used predicted amino acid sequences and neighbor‐joining method implemented in MEGA7 22 with 1000 bootstrap replicates.

### Samples

**Figure 1:**
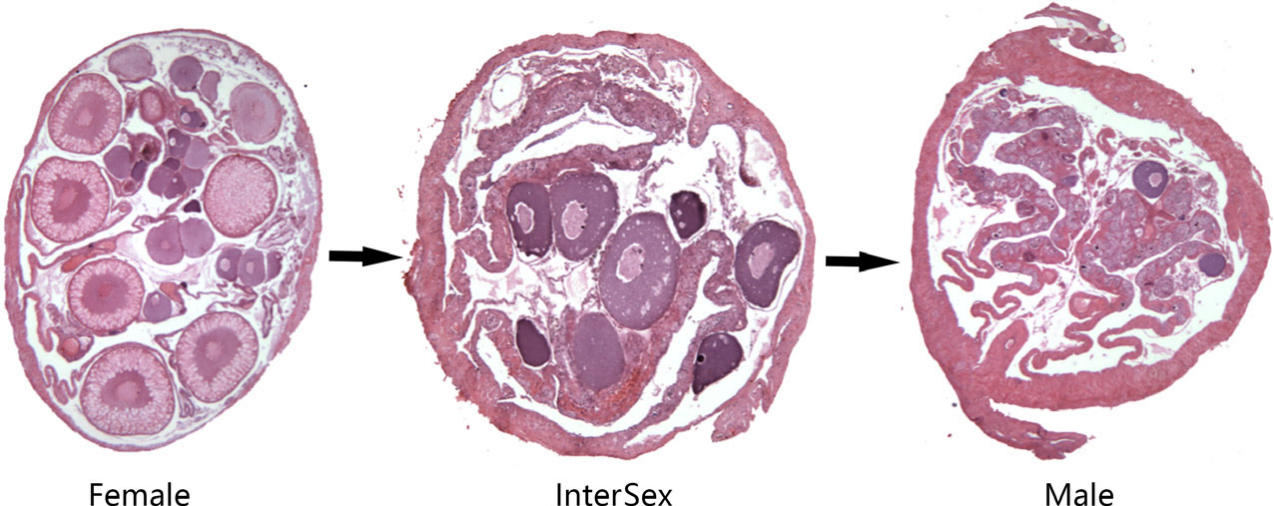
Microscopic images of gonads (female, intersex, and male).

Specimens used for experiments were hatched and cultured at the breeding base of the Hubei Provincial Engineering and Technology Research Center for Asian swamp eel. Female swamp eels usually enter the intersex stage immediately after spawning (laying eggs); the intersex period, characterized by simultaneous ovarian degeneration and testicular development, lasts for about 2–3 months, after which the transition into males is complete. Generally, 1‐year‐old swamp eels are all females; among the 2‐year‐olds, 70% are females and 30% are males; and among the 3‐year‐olds, 90% are males and 10% are females. For the experiment, we selected three (apparently) healthy specimens of each sex: females aged ~ 12 months, intersex specimens ~ 18 months, and males ~ 36 months (Table 1). Specimens were euthanized in buffered MS‐222 (400 mg·L^−1^ concentration), immediately dissected, and the following tissues collected: liver, muscle, spleen, kidney, intestines, heart, gonads, brain, and pituitary gland. Tissues were immediately macrodissected into several ~ 0.1 g sections (to be used for RNA extraction directly), flash‐frozen in liquid nitrogen, and stored at −80 °C until RNA extraction. To corroborate the sex, a section of gonads was taken for histological examination, conducted under an electronic microscope as described 23, 24. Under the electron microscopy, only oocytes can be seen in females, oocytes and sperm cells can be seen in intersexuals, and only sperm cells can be seen in males (Fig. 1). All animals were handled and experimental procedures conducted in accordance with the guidelines for the care and use of animals for scientific purposes set by the EU Directive 2010/63/EU. Animal experiments for this study have been reviewed and approved by the Ethics Committee of the Yangtze University.

**Table 1:** Total length (TL, cm) and weight (*W*, g) of *Monopterus albus* specimens used for qPCR experiments.

Specimen No.	Females		Intersex		Males	
	TL	*W*	TL	*W*	TL	*W*
1	15	40	28	86	30	101
2	18	45	28	88	32	103
3	19	48	29	85	36	105
Average	17.33	44.33	28.33	86.33	32.67	103.00
SEM	2.08	4.04	0.58	1.53	3.06	2.00

### RNA, cDNA, and RACE

**Figure 2:**
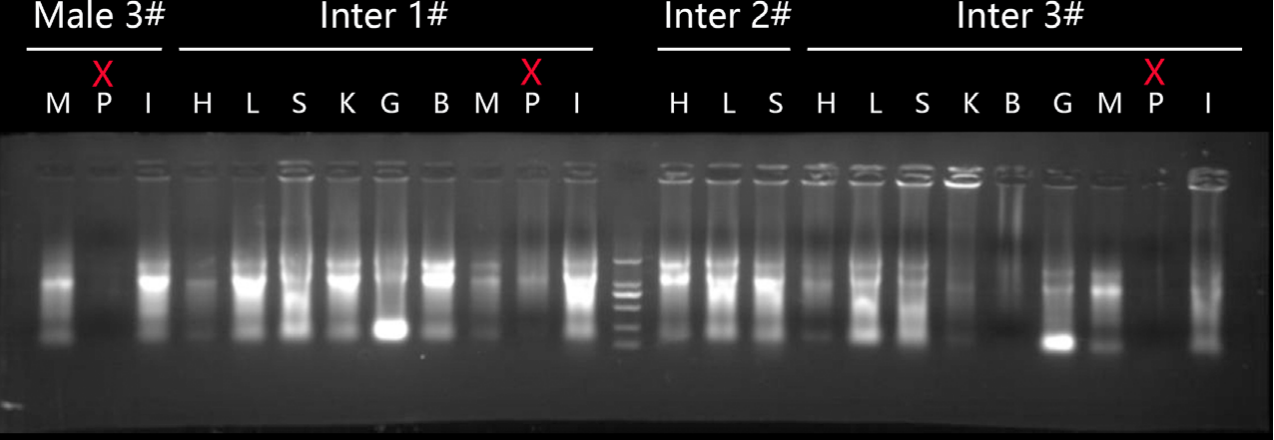
Electrophoresis of extracted RNA. Inter is intersex, H is heart, L is liver, S is spleen, K is kidney, B is brain, G is gonad, M is muscle, P is pituitary gland, and I is intestine. Discarded samples are highlighted with a red X sign above.

Samples (~ 0.1 g) were ground in liquid nitrogen with mortar and pestle, and RNA was extracted following the standard TRIzol extraction protocol. Quality and quantity of the extracted RNA were assessed by electrophoresis on 1% agarose gel (Fig. 2) and NanoDrop 2000 spectrophotometer (Thermo Scientific, Waltham, MA, USA). cDNA library was synthesized using RevertAid^TM^ First Strand cDNA Synthesis Kit with DNase I (#K1621 and #EN0521, respectively, both Thermo Scientific) following the manufacturer’s protocol, and cDNA stored at −80 °C. As we could identify only one *kiss*/*gpr54* gene using the methodology described in the first section, to further confirm that there are no other expressed *kiss*/*gpr54* paralogues present in the transcriptome of *M. albus* (most importantly, to corroborate that *kiss1* and *gpr54‐1* are absent), we designed degenerate primers for *kiss1*, *kiss2*, and *gpr54* genes (Table 2) using a number of orthologues from closely related teleost species and used them to amplify transcripts present in the transcriptome (cDNA). The PCR mix (50 µL) contained: Ex Taq (Takara, Beijing, China) 1.0 μL, 2× Ex Taq Buffer (Takara) 25.0 μL, dNTP Mix (10 mm), 1.0 μL primers (10×) 1.5 μL each, cDNA 5.0 µL, primers (10×) 1.5 μL each, PCR‐Grade Water 15.0 µL. Conditions were as follows: denaturation 94 °C 2 min, 35 cycles 94 °C 30 s, 55 °C 30 s, 72 °C 1 min, followed by 72 °C 10 min. Only a single band was observed for both genes after PCR products were electrophoresed. The bands were immediately cut out and DNA recovered using a Glue Recovery Kit (OMEGA, Omega Bio‐tek Inc., Norcross, GA, USA) according to the manufacturer’s instructions. After cloning and sequencing (as described below), these segments were used to design primers for RACE PCR, conducted with the aim to clone full‐length *kiss* and *gpr54* mRNA sequences from the cDNA library synthesized using RevertAid^TM^ First Strand cDNA Synthesis Kit (Thermo Scientific; 5′ RACE) and SMARTScribe Reverse Transcriptase (TaKaRa; 3′ RACE) following the manufacturer’s protocols. Primers for RACE PCR (Table 2) were designed using Primer Premier (Biosoft, Palo Alto, CA, USA) and synthesized by Shengwu Gongcheng Co. Ltd. (Shanghai, China). Both 5ʹ and 3ʹ RACE PCR were conducted using SMARTer RACE cDNA amplification kit (Takara), following the protocol. PCR products were purified using the Gel Extraction Kit (Tiangen Biotech, Beijing, China) and then ligated into a PMD18‐T vector (TaKaRa), transformed into competent *Escherichia coli* DH5α cells, plated on a Petri dish containing LB‐agar supplemented with ampicillin (100 μg·mL^−1^) for selection, and incubated overnight at 37 °C. Colony PCR was used to screen positive colonies, three of which were picked for sequencing.

**Table 2:** Primers used in this study. *T*_M_ is melting temperature; Amp is the size of the amplicon; and reference indicates a reference gene for qPCR.

Gene	Note	Name	Sequence	*T*_M_	Amp
*kiss1*	Degenerate	F	TGGCTRCTTYGTCARCAGA	49.03	287
		R	TCATTTYCCRTAACGKARACC	54.42	
*kiss2*	Degenerate	F	TGGCTBTGGYBGTKGTBTG	48.51	241
		R	GGGTTGWAGTTGAAHTTRCT	49.98	
*gpr54*	Degenerate	F	ATGTACTCCTCYGAGGARCT	54.50	946
		R	CRTAGGACATGCAGTTGGCC	58.02	
*kiss2*	qPCR	F	GAGAGAATGAAGACCAGCGG	58.07	157
		R	CGCGTGAAGAGAGAAATGGG	59.00	
*gpr54*	PCR	F	ATGTACTCATCTGAAGAACTGTGG	57.4	1134
		R	TCAATTCACTCCGTTGTTATTG	57.70	
*gpr54*	qPCR	F	TTGGGTCCTTCATCCTGTCC	62.18	143
		R	CGCGATGAACTGGTAGAGGA	61.34	
*β‐actin*	Reference	F	CTGGACTTCGAGCAGGAGAT	58.89	144
		R	ACCAAGGAAAGAAGGCTGGA	58.85	
*GAPDH*	Reference	F	CTTTGGCATCGTTGAGGGTC	62.86	124
		R	AGGGATGATGTTCTGGCTGG	52.43	
*kiss2*	5′RACE	GSP1	TCCACCAGGATGTCTC	49.52	
		GSP2	GTTGTCCTCCTGAGCG	53.01	
		GSP3	ACTGATCCTGTTGCTCGC	57.38	
	3′RACE	C032‐1	CATCAAAATGGAGCCGCCGTGTCA	73.66	
		C032‐2	GCCGTTGCAGCCCAATTTTATCATC	69.93	
*gpr54‐2*	5′RACE	GPR1	GGTGCTGATCTTCCTC	43.00	
		GPR2	AAGTTCGCCTCTGAGCCA	49.59	
		GPR3	TCATTGTCTCTATGTTTTCC	56.53	
	3′RACE	C032‐3	CCTGTTCAAGCACAAGGTCAGAGAC	57.66	
		C032‐4	GCTTCAAGGACTGCCAACGCTGAG	60.59	

### qPCR

cDNA was diluted twofold and used as a template for qPCR, which was conducted on CFX Connect™ (Bio‐Rad, Hercules, CA, USA), using Power SYBR® Green PCR Master Mix (Applied Biosystems®, Waltham, MA, USA Cat: 4367659). Cycling conditions were as follows: 95 °C for 3 min, followed by 40 cycles of 95 °C for 10 s, 55.0 °C for 20 s, 72.0 °C for 20 s, and plate reading at 75 °C for 5 s. qPCR specificity was assessed using melt curves (65–95 °C, with 0.5 °C increments) and electrophoresis. Previous studies in this species mostly relied on *β‐actin* as the reference gene 25, 26, but we also tested the expression of another commonly used reference gene, *GAPDH*, in different tissues. Stability of Ct values of these two genes was analyzed using the comparative cycle threshold method implemented in the GeNorm software 27. Although both were suitable, *β‐actin* exhibited less variability (Table S1). Gene expression levels were calculated using the 2^−ΔΔCT^ method 28. As we could not obtain results for some samples (difficulties during RNA extraction), some results are based on three and some on two biological replicates (Table S1). Blanks were included in each run, and technical replicates were triplicate. qPCR experiments were conducted by the Bio‐Transduction Lab (Wuhan, China). MIQE checklist is available as Table S2.

## Results

### Identification and characterization of *kiss2* and *gpr54* genes in *M. albus*

**Figure 3:**
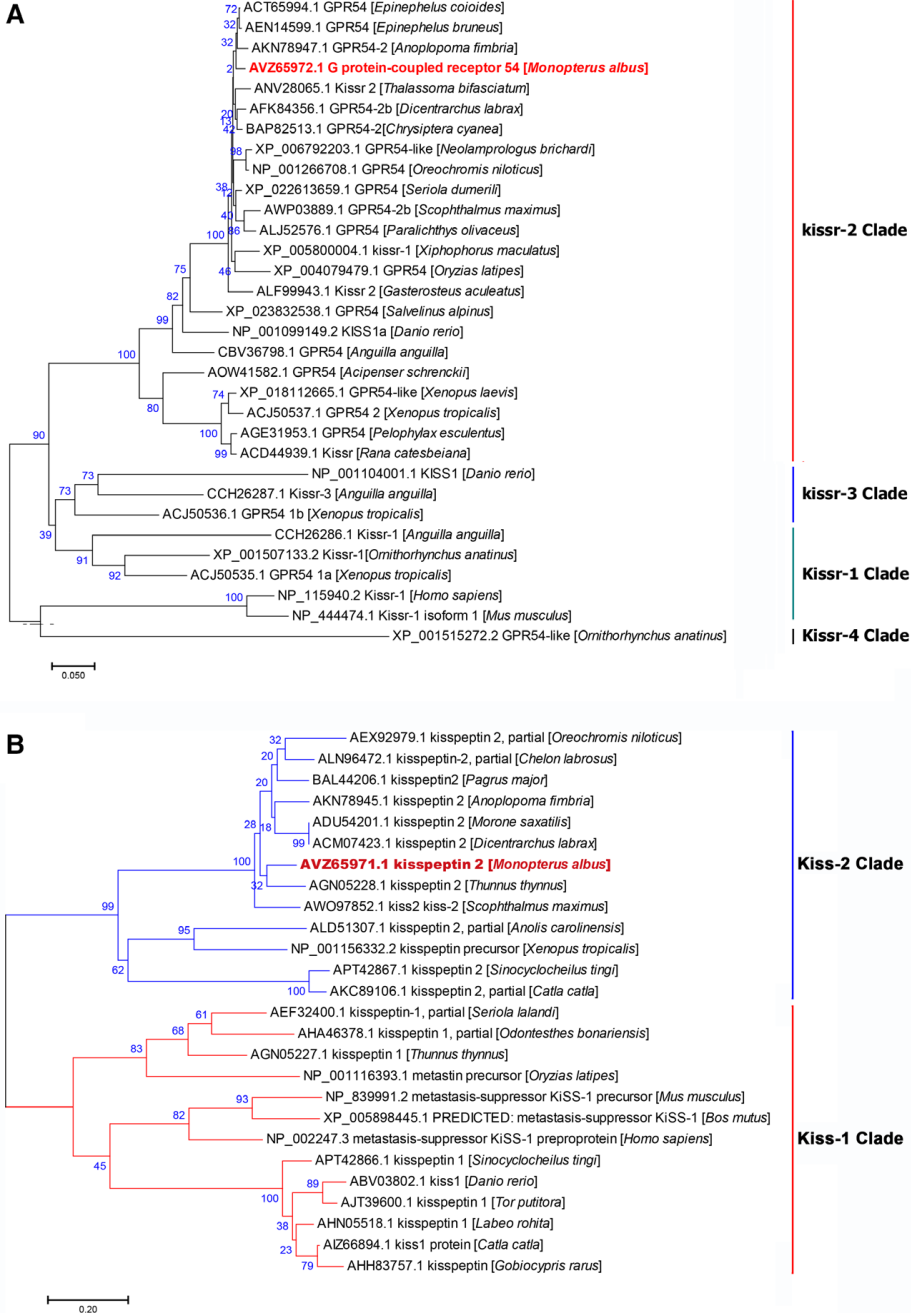
Phylogenetic analyses of the identified *Monopterus albus kiss2* (A) and *gpr54‐2* (B) genes. A number of vertebrate *kiss* and *gpr54* homologues were selected for the analysis. Gene families are indicated to the right. GenBank accession numbers and names are shown in the figure.

**Figure 4:**
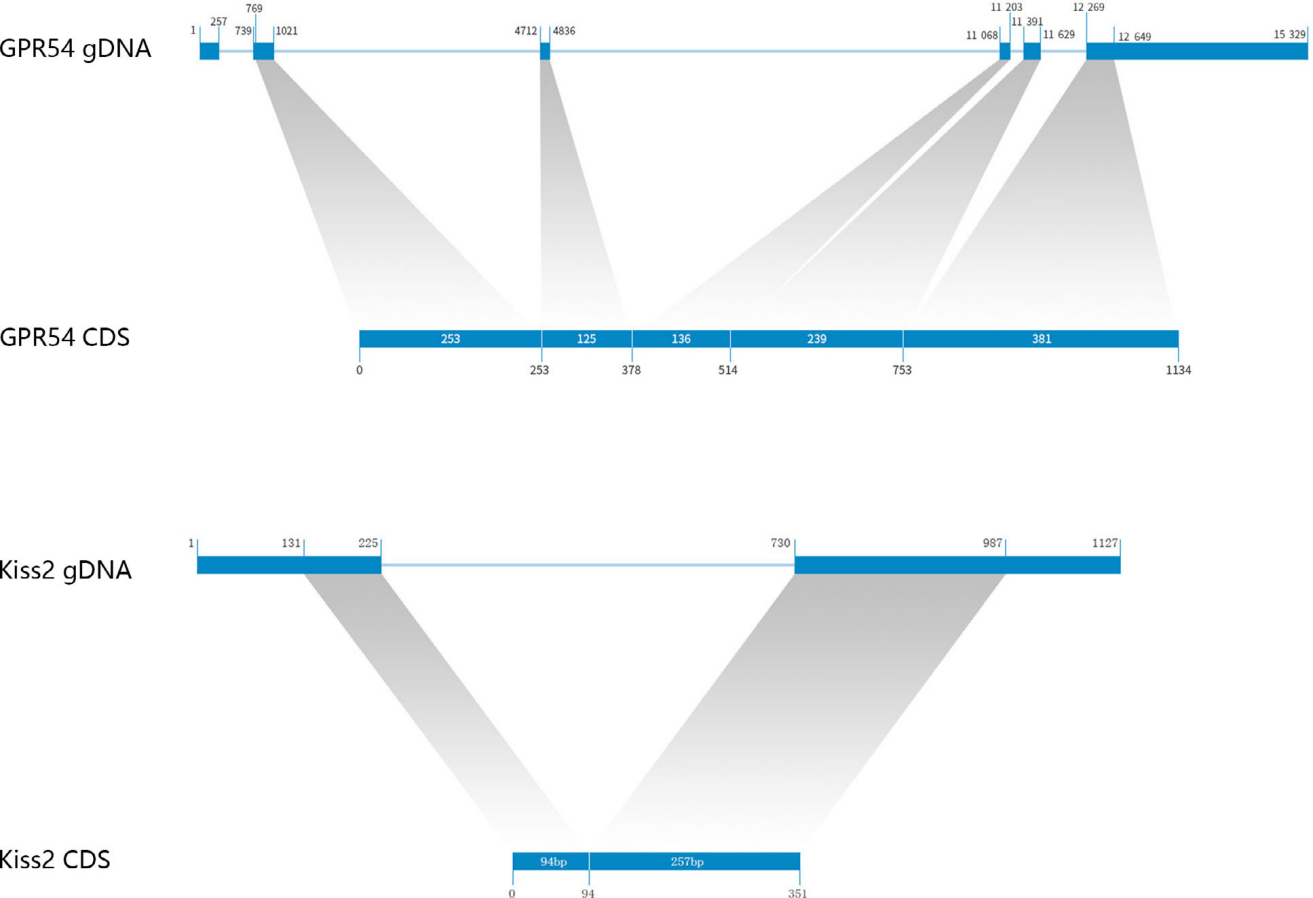
*gpr54* and *kiss2* size and organization: nuclear genome (including intron/exon) and expressed CDS.

We identified only one copy of each of the two genes in the genome (and transcriptome) of *M. albus*. *Kiss2* was identified within the scaffold ID NW_018127972.1 (positions 3063234–3063734, identity 100%). *Gpr54* was identified against the annotated sequence of this gene (XM_020591446, LOC109955350, identity 99%: 1131/1134 bp). We did not find any indications of the existence of splice variants in our sequencing results. Phylogenetic analyses corroborated that they are orthologues of *kiss2* and *gpr54‐2* (or *kissr2*) genes (Fig. 3). The *kiss2* mRNA sequence is composed of 656 nucleotides (CDS = 351 nucleotides), encoding a protein of 116 amino acids. It contains putative Kisspeptin‐10 (FNLNPFGLRF) and Kisspeptin‐12 (SKFNLNPFGLRF) fragments 10. *gpr54‐2* is composed of 1134 bases, encoding protein of 377 amino acids. *kiss* CDS comprised two exons and *gpr54* CDS comprised five exons (Fig. 4). Sequences are deposited in GenBank under the accession numbers MF085053 (*kiss2*) and MF085054 (*gpr54*).

### Expression patterns of *gpr54* and *kiss2* genes

**Figure 5:**
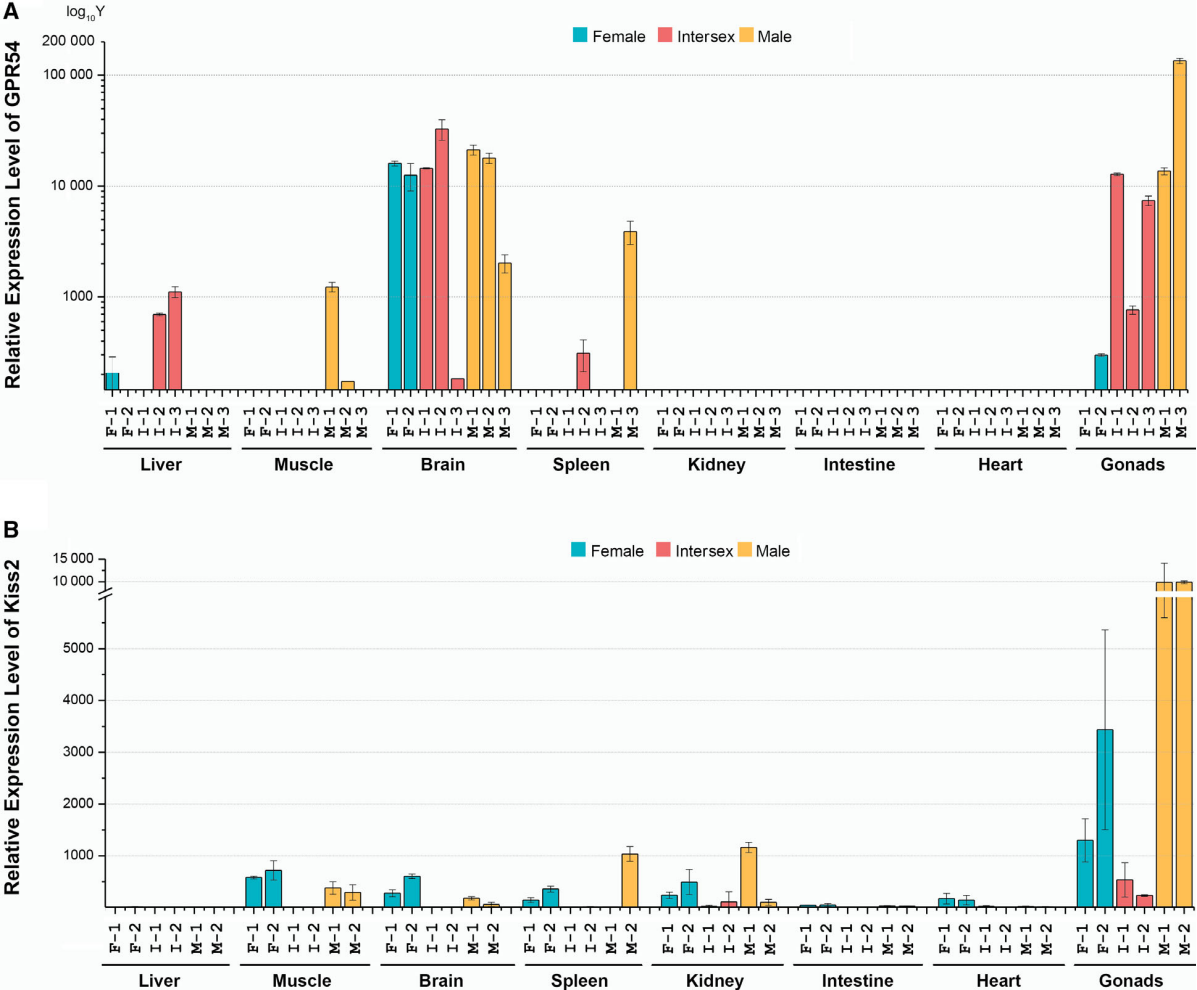
Expression patterns of *kiss2* (A) and *gpr54* (B) genes in tissues of female, intersexual, and male *Monopterus albus*. mRNA expression levels were analyzed by qPCR and presented as expression in a single specimen (average of three technical replicates). All expression levels are normalized to the average expression in a single intersex sample: *gpr54* to the I‐3 muscle and *kiss2* to the I‐1 intestine (samples are presented as ‘sex – sample number’, so I‐3 stands for ‘intersex specimen no. 3’). Error bars represent SEM of three technical qPCR replicates.

RNA extraction from pituitary gland was not successful (Fig. 2), so the expression was studied in liver, muscle, brain, spleen, kidney, small intestine, heart, and gonads of male, intersex, and female specimens (Fig. 5; Table S1). *Gpr54* was very highly expressed in the brain of all three sexes (> 10 000‐fold), but M‐3 sample (male‐3) was a minor outlier, and I‐3 was a major outlier (Fig. 5A). The highest expression among all samples was observed in male gonads: > 100 000 000‐fold in the M‐3. Expression was also very high in the intersex gonads (~ 10 000‐fold), while expression in female gonads was much lower (< 500‐fold). Its expression levels were comparatively low in all other tissues, with the following minor exceptions: male spleen (M‐3 sample ~ 3000‐fold, but very low in the M‐1 sample), intersex liver (intermediate expression of 700‐ to 1000‐fold, but very low in the I‐1 sample), and male muscle (low in M‐2 and M‐3, but M‐1 was an outlier with > 1000‐fold).

*Kiss2* was exceptionally highly expressed in the gonads of males (both samples ~ 10 000‐fold; Fig. 5B). Expression in female gonads was also comparatively high (> 1000‐fold, but with rather high variability among technical replicates), but in the gonads of intersex specimens, its expression was much lower (< 600‐fold). Expression in all other tissues was comparatively low, with a minor exception of the female muscle (~ 600–700‐fold), and two individual outlier samples: M‐2 spleen and M‐1 kidney (both > 1000‐fold). Intriguingly, expression in the brain tissue of all three sexes was also very low: ~ 300–600‐fold in females, ~ 60–180‐fold in males, and ≤ 1‐fold in intersex.

## Discussion

Although the ability to manipulate the timing of puberty and reproductive development in farmed fish is of major commercial relevance 16, the understanding of sex reversal phenomenon in fishes is important and fascinating from a much wider biological, ecological, and evolutionary perspective 2. A number of gene (and genome) duplications (and subsequent gene losses) in the evolutionary history of fish 10, 11, 29, 30 and the existence of compensatory mechanisms 19 make the studies of kiss/gpr54 system in fish very complex. Both genes (*kiss2* and *gpr54‐2*) identified in the genome of *M. albus* are comparable to those characterized in other teleost fishes in terms of size 12, 14, 18. There is evidence that *kiss1* and *kiss2* have different functions in other fish species 10, 11, 14; for example, in medaka (*Oryzias latipes*), only the *kiss1* system, but not the *kiss2* system, shows expression dynamics strongly indicative of its direct involvement in the HPG axis regulation 17. As opposed to this, in some other fishes, which includes *M. albus*, the *kiss1* gene is missing, and only *kiss2* is expressed 17. As the evolution of Kisspeptin and its receptor in fish, aside from gene duplications and loss, involves appearance of splice variant isoforms as well 10, we hypothesized that the decreased size of the genome of *M. albus* may have resulted in a ‘space‐saving’ functional adaptation: replacing a paralogue with an isoform. However, after searching the genome, transcriptome, and proteome of *M. albus*, we could not find evidence for the existence of paralogues or isoforms, which indicates that *kiss1* may have been functionally replaced in *M. albus* with *kiss2* or some other compensatory mechanism.

Constitutive expression of *kiss2* and/or *gpr54‐2* in all studied tissues has been observed in several fish species 12, 14, 16. Although we detected both genes in all studied tissues, their expression levels were generally low, and in some cases barely detectable. An intriguing observation regarding the constitutive expression pattern of *kiss2* is a notably lower expression in intersex specimens (barely detectable in most tissues apart from gonads, where it was also much lower than in males and females). This indicates that *kiss2* appears to undergo a ‘reset’ in the expression as *M. albus* undergoes the sex change. This might be in correlation with the proposed important role of the kisspeptin system in modulating gonadal sex differentiation, pubertal timing, and reproduction in teleost fishes 17, 18. In the light of these findings, it would be of interest to assess the constitutive expression of *kiss2* in juvenile (prepubescent) *M. albus* in future studies.

Both genes are generally highly expressed in fish brain 12, 14, 16, 18, 31, 32, where they probably have a broad range of functions 33, 34, 35. The expression of *gpr54*‐*2* in the brain of *M. albus* is in full agreement with this, with similar (high) expression levels among all three sexes, which suggests that functions in brain do not vary between sexes. This may be a reflection of the notorious promiscuousness of kiss receptors, which is believed to be an evolutionary guarantee of functional robustness of the HPG axis regulation system in cases of gene loss or neofunctionalization 17. *Kiss2*, however, was not highly expressed in any of the studied brain samples, which may be an indication of functional diversification. However, as the expression of these two genes varies strongly among different parts of the brain 12, 14, 36, so this may also be a reflection of the fact that we sampled (almost) entire brains, and failed to extract DNA from the pituitary gland, which may have skewed the overall expression results downwards.

Expression patterns of all of these genes (including paralogues) are generally extremely variable, both among tissues and paralogues themselves 18, but it remains unclear whether this is merely a reflection of molecular noise 37, or of species‐specific functions of different paralogues. For example, patterns differed among all four genes (two sets of paralogues) in chub mackerel, with the *kiss2* gene expressed only in the brain, and neither of the two kiss‐receptor genes expressed in the ovary 18, which is notably different from our results. This is not surprising, as they are likely to be functionally distinct: Multiple studies have shown that in fish *kiss1* appears to be more effective than *kiss2* at inducing puberty 18, but *kiss2* has a stronger stimulatory effect on the testicular development during nonbreeding period 38. This is in agreement with high *kiss2* expression in the gonads of male *M. albus*. Overall, studies suggest that kisspeptin peptide modulates gonadotropin secretion and influences gonadal development in fish, but the effects on ovarian development appear to be slower than the effects on testicular development 18. This may bear importance for the interpretation of our results, as the expression in female gonads was much lower than the expression in male gonads.

In zebrafish, where two *gpr54* paralogues have been identified, *gpr54‐1* was expressed in the brain and gonads, while *gpr54‐2* was constitutively expressed in most tissues 32. The expression pattern of *gpr54‐2* in *M. albus* therefore appears to resemble that of zebrafish *gpr54‐1*, but only in males. Most importantly, *gpr54‐2* expression pattern in gonads (extremely high in males, high in intersex, low in females) presents a strong evidence of sex‐specific expression pattern of this gene in the gonads of *M. albus*.

Unfortunately, the interpretation of our data is hampered by technical difficulties that we experienced during the RNA extraction, which resulted in us having to discard some samples. This in turn resulted in a small number of biological replicates (fish specimens) used in the study and prevented us from conducting meaningful statistical analyses. Furthermore, some of our samples also exhibited signs of RNA degradation (Fig. 2), so it will be necessary to have our findings corroborated on a larger number of samples by a future study. Difficulties in the extraction of RNA from fish gonads have been discussed in detail before 39, so future studies should also probably try to tinker with their RNA extraction protocols in order to obtain a higher‐quality RNA.

## Conclusions

Using *M. albus* as a model, this study aimed to improve the understanding of Kisspeptin/GPR54 axis functioning in the sex reversal in fish (and vertebrates in general). The main conclusions that can be inferred from our results are summarized as follows: Kiss1 system (present in many other fish species) has been functionally replaced in *M. albus* with Kiss2 system or some other compensatory mechanism; expression of *gpr54‐2* in brain does not appear to vary between sexes; *kiss2* expression appears to undergo a ‘reset’ in expression as *M. albus* undergoes the sex change; and we found a strong evidence of sex‐specific expression pattern of both *kiss2* and *gpr54‐2* in the gonads of *M. albus*. Although these findings indicate that Kisspeptin/GPR54 system might indeed play a role in the sex reversal of *M. albus*, interpretation of these data and comparisons between species are rendered exceptionally complex by the existence of multiple paralogues in other fish species, by their species‐specific functions, diverse reproductive strategies, and differences in experimental approaches between studies 10, 16. Additionally, as targeted mutations of *kiss* and *gpr54* do not disrupt gonadal development and reproductive performance in zebrafish, this is a strong indication of the existence of a compensatory mechanism that ensures reproductive success 19, 40, 41. This suggests that, as opposed to mammals, kisspeptin may not be a central and absolute upstream regulator of the HPG axis in fish 18. Regardless of these limitations, in future studies we aim to explore the relationship between the regulation of these genes and downstream sex reversal and gonadal differentiation‐associated genes and hormones, and thereby improve the understanding of Kisspeptin/GPR54 axis functioning in sex reversal in fish using *M. albus* as a model.

## Conflict of interest

The authors declare no conflict of interest.

## Author contributions

TLY and DQY conceived and designed the project; TLY and MTP acquired, analyzed, and interpreted the data; and TLY and DQY wrote the paper.

## Supporting information


**Table S1**. qPCR data.

**Table S2**. MIQE checklist.

## Data Availability

The two sequenced genes are deposited in GenBank under the accession numbers MF085053 (*kiss2*) and MF085054 (*gpr54*).
